# Radioiodine therapy under rhTSH for follicular thyroid carcinoma with sellar metastasis and non-rising TSH

**DOI:** 10.1259/bjrcr.20150322

**Published:** 2016-05-15

**Authors:** Sameer Kamalakar Taywade, Nishikant Avinash Damle, Kunal Kumar, Piyush Ranjan, Shipra Aggarwal, Chandrasekhar Bal, Amandeep Kumar, Devasenathipathi Kandasamy

**Affiliations:** ^1^ Department of Nuclear Medicine, A.I.I.M.S, New Delhi, India; ^2^ Department of Surgery, A.I.I.M.S, New Delhi, India; ^3^ Department of Pathology, A.I.I.M.S, New Delhi, India; ^4^ Department of Neurosurgery, A.I.I.M.S, New Delhi, India; ^5^ Department of Radiodiagnosis, A.I.I.M.S, New Delhi, India

## Abstract

Metastasis to the pituitary gland/sella turcica is an uncommon complication of thyroid cancer. Treating this condition is a challenge in the setting of pituitary insufficiency due to this lesion, and recombinant human thyroid-stimulating hormone (rhTSH) stimulation becomes critically essential. We present a rare case of an 82-year-old female patient with follicular carcinoma of the thyroid with metastasis to the sella turcica in addition to multiple skeletal and lung metastases. MRI of the brain showed a hypointense suprasellar lesion on *T*_1_ weighted images. The thyroid-stimulating hormone level remained persistently low even 4 weeks after thyroidectomy. A whole-body pertechnetate scan could not localize any abnormal tracer uptake and radioactive iodine uptake was also persistently low. The patient did not have symptoms related to pituitary involvement but TSH and early morning adrenocorticotrophic hormone levels were low. After thorough discussion with the neurosurgeon and radiotherapist, it was decided to start the patient on high-dose radioiodine treatment. Persistently low TSH level was a concern for starting radioiodine therapy. In view of this clinical context, rhTSH stimulation was used to achieve adequate TSH levels prior to radioiodine therapy. Subsequently, the patient was treated with 3.7 GBq (100 mci) of high-dose radioiodine. A post-therapy scan demonstrated radioiodine concentration in the thyroid bed remnant, multiple skeletal lesions and the sellar region. Thus, the use of rhTSH was critical in the management of this patient. It helped in radioiodine treatment by stimulating radioiodine uptake in the remnant and at the metastatic sites.

## Case report

**Figure 1.**
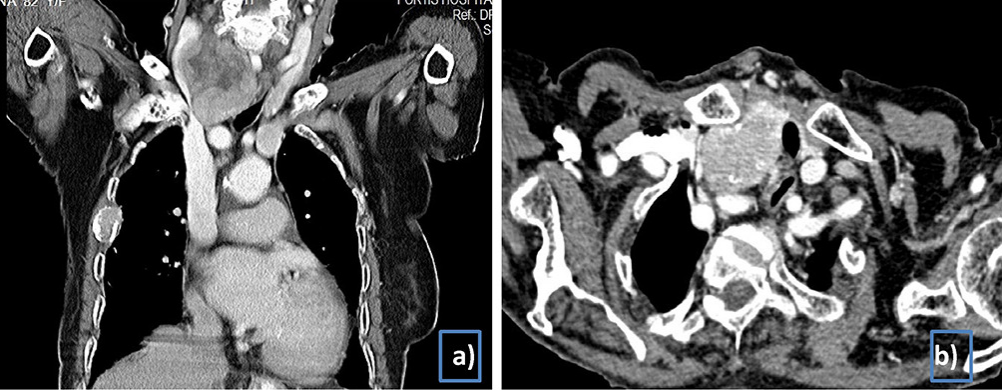
(a, b) Pre-operative contrast-enhanced CT scan of the neck and chest. A large enhancing mass lesion with central necrosis in the right lobe of the thyroid causing compression and deviation of the trachea to the left side.

An 82-year-old female patient presented with a history of goitre for 10 years with recent rapid increase in its size, dysphagia and hoarseness of voice for 1 month. She also had low backache and swelling over the right parietal region for 5 months. She had tenderness over the lower lumbar spine on examination. Palpation revealed a large 10 × 8 cm firm mass in the anterior neck, predominantly on the right side, and an immobile hard swelling over the scalp in the right parietal region. Contrast-enhanced CT imaging (Figure 1) performed previously showed a large enhancing mass involving the right lobe and isthmus of the thyroid gland with multiple subcentimetre nodules in bilateral lungs. Fine needle aspiration cytology of the thyroid mass revealed it to be a follicular neoplasm (Bethesda category IV). Based on this clinical information, a tentative diagnosis of metastatic follicular thyroid carcinoma was made. The patient was planned for thyroidectomy followed by radioactive iodine therapy depending on the histopathology. She underwent right hemithyroidectomy in December 2014 under cervical block. Intraoperatively, the surgeon visualized a very small atrophic left lobe of the thyroid but in view of the patient's overall condition, complete thyroidectomy was considered a difficult procedure to perform. The final histopathology report revealed follicular carcinoma with capsular and vascular invasion.

**Figure 2.**
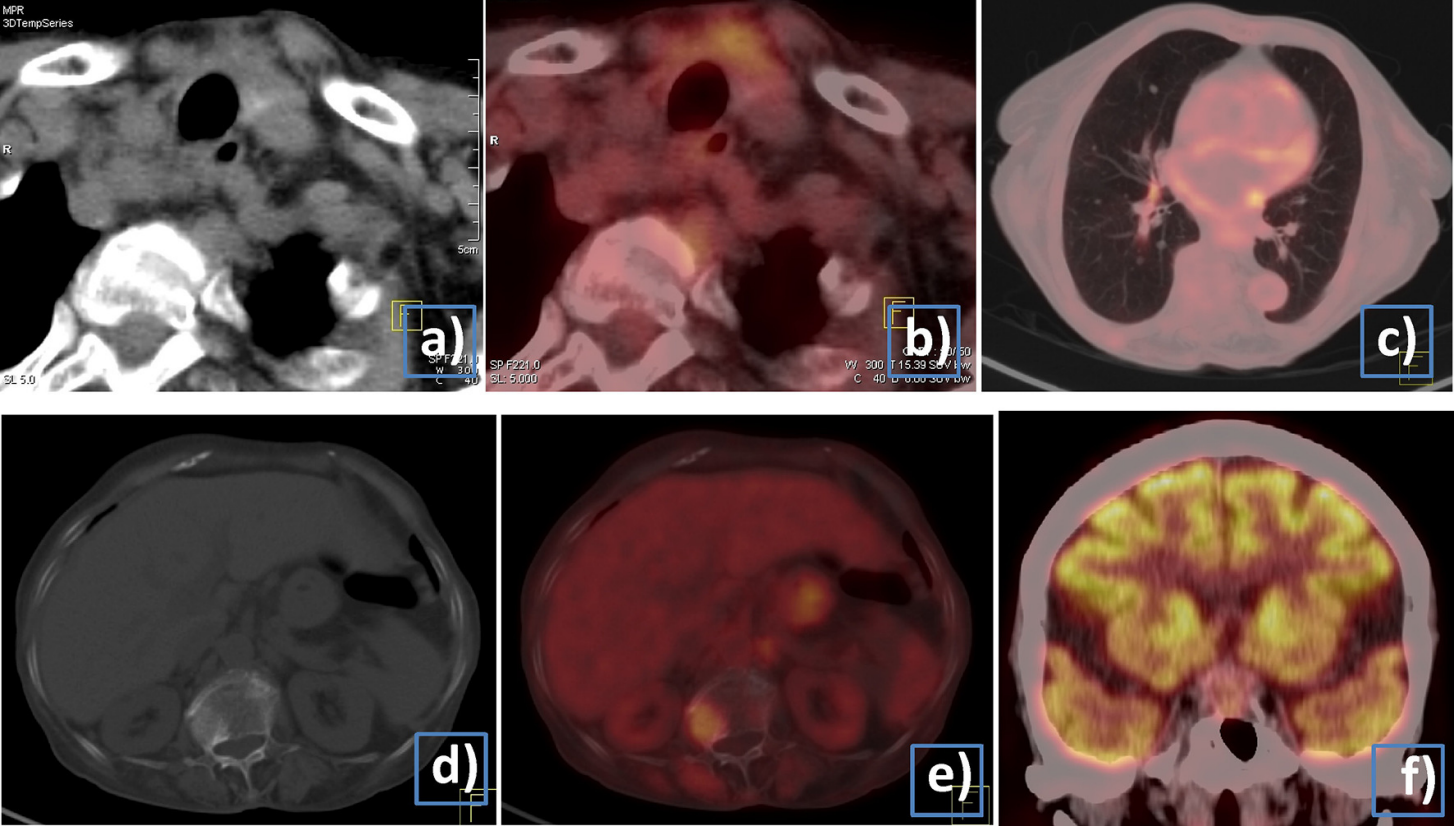
(a–f) ^18^F-FDG PET-CT. (a, b) CT and fused ^18^F-FDG PET-CT transaxial images showing residual thyroid tissue in the neck, predominantly on the left side; (c) fused ^18^F-FDG PET-CT transaxial image shows multiple small lung nodules; (d, e) CT and fused ^18^F-FDG PET-CT transaxial images show a sclerotic lesion in the vertebra with mild FDG uptake; (f) coronal ^18^F-FDG PET-CT fused image shows minimal FDG uptake in the sellar lesion. ^18^F-FDG PET-CT, 18-fludeoxyglucose positron emission tomography-CT.

**Figure 3.**
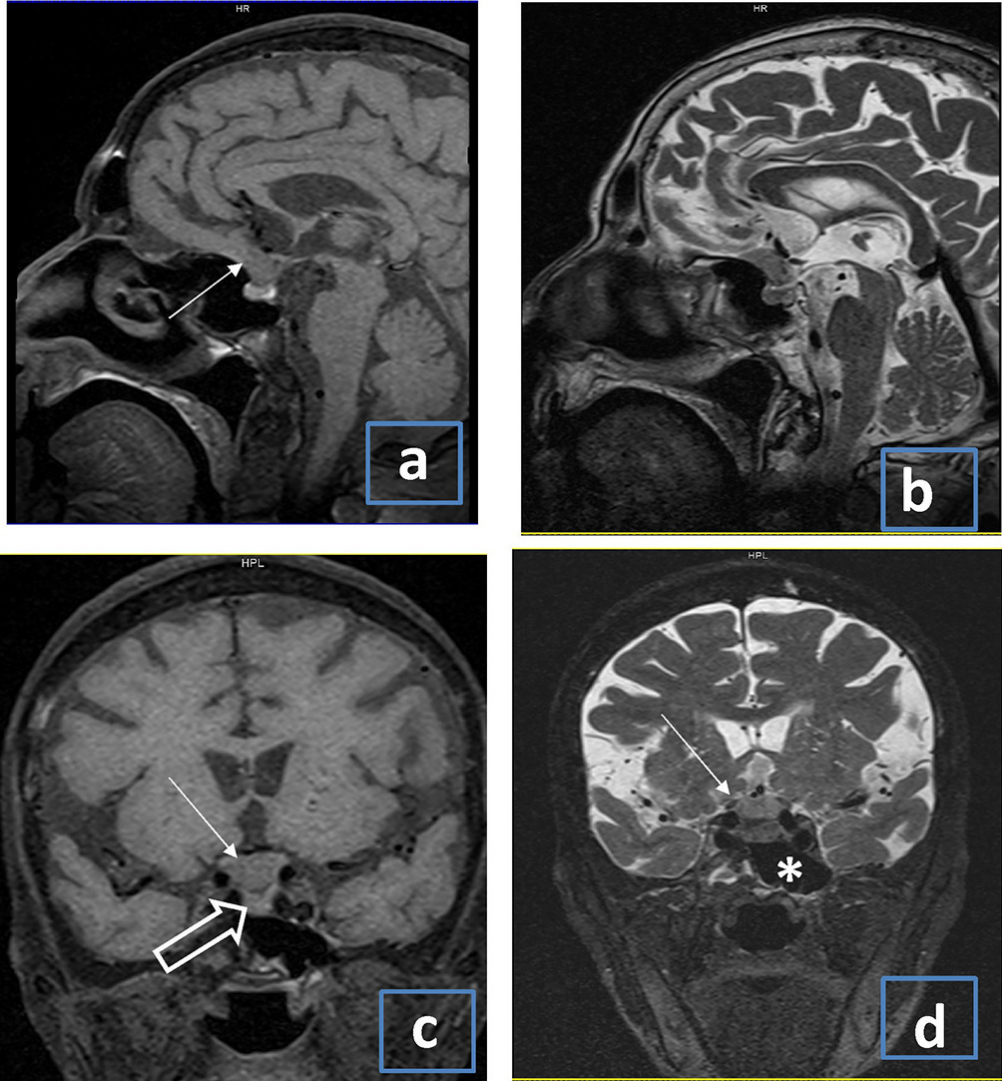
(a–d) MRI of the brain. *T*_1_ weighted image in the sagittal plane (a) showing a mildly hypointense lesion (arrow) based over the sphenoid bone seen in the suprasellar region. The lesion is isointense on sagittal *T*_2_ weighted image (b) and does not show any bleeding or necrosis within. *T*_1_ and *T*_2_ weighted images in the coronal plane (c, d) showing the lesion (arrows) in the suprasellar region with sparing of normal pituitary (outlined arrow). The sphenoid sinus (asterisk) is also normal.

**Figure 4.**
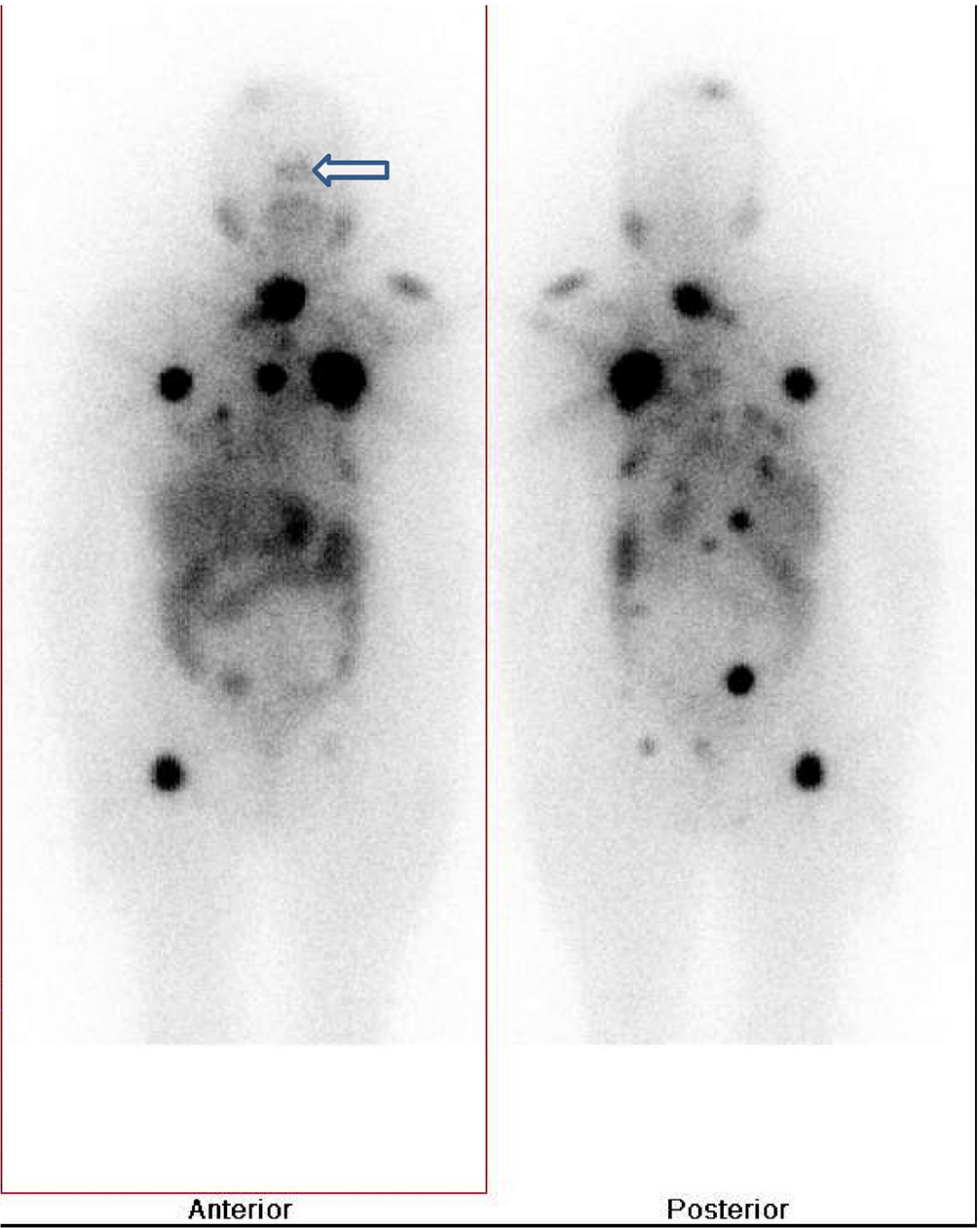
Post-therapy I-131 whole body scan. Anterior and posterior images show a remnant in the thyroid bed with intense iodine uptake, multiple iodine-avid skeletal metastases and mild iodine uptake in the lesion in the sellar region (outlined arrow).

The patient was referred to the department of nuclear medicine for further management. As per institutional protocol, after hemithyroidectomy, she underwent radioactive iodine uptake (RAIU) and thyroid scan, which showed 1.4% 24 h RAIU and no pertechnetate uptake in the region of the thyroid gland. A pertechnetate whole-body sweep was performed in the same sitting and revealed no uptake in the already known metastatic site. Thyroid function test showed a low normal thyroid-stimulating hormone (TSH) value (0.5 µIU ml^–1^) with normal T3 and T4 levels. Neck ultrasonography showed residual tissue in the neck, predominantly on the left side. In view of the history of a contrast-enhanced CT scan 4 weeks ago, the possibility of iodide interference was considered. The patient was given tablets of furosemide 40 mg day^–1^ for 2 weeks and a repeat RAIU and pertechnetate study was planned after 2 weeks. The repeat pertechnetate scan did not show abnormal tracer accumulation anywhere in the body and no uptake in the thyroid bed, and there was persistently low RAIU at both 2 (1.5%) and 24 h (1.4%). Repeat thyroid testing showed that the TSH level was 0.5 µIU ml^–1^, while T3 and T4 levels were now lower than normal. Thyroglobulin level post hemithyroidectomy was reported as > 300 ng ml^–1^ (normal range 0–52 ng ml^–1^) by chemiluminescence. The progressively decreasing total T3 and T4 levels ruled out the possibility of extensive functioning metastasis. The possibility of metastases from a second primary was also considered in view of the absence of iodine uptake in the lung and bone lesions. Whole-body ^18^F-fludeoxyglucose positron emission tomography–CT (^18^F-FDG PET-CT) scan (Figure 2) demonstrated significant residual thyroid tissue in the thyroid bed, multiple skeletal metastases with mild FDG uptake and bilateral pulmonary nodules. In addition to this, there was a small iso- to hyperdense lesion in the sellar region with mildly increased FDG uptake. Furthermore, MRI of the brain (Figure 3) showed a hypointense lesion on *T*_1_ weighted images and isointense lesion on *T*_2_ weighted images based over the sphenoid bone in the suprasellar cistern with no bleeding or necrosis. The pituitary gland was seen separately. The right parietal lesion seen in both ^18^F-FDG PET-CT scan and MRI showed only dural involvement with no infiltration of adjacent brain parenchyma. The MRI and ^18^F-FDG PET-CT findings pointed toward the possibility of pituitary insufficiency owing to sellar metastasis resulting in low TSH. To evaluate the pituitary insufficiency further, serum cortisol and adrenocorticotrophic hormone (ACTH) assay was performed. Early morning (4 am) ACTH was < 5 pg ml^–1^ (10–60 pg ml^–1^) and cortisol was 1.16 µg ml^–1^ (4.3–22.4 µg ml^–1^). This reconfirmed the diagnosis of pituitary insufficiency due to sellar metastasis with low iodine and pertechnetate uptake owing to the low TSH level. The treatment option in this clinical context was discussed thoroughly with a neurosurgeon and the radiotherapist. The patient had neither diabetes insipidus nor symptoms of cranial nerve involvement. In view of the extensive metastases and no significant neurological manifestation, surgery, external beam radiotherapy (EBRT) and stereotactic radiosurgery were not considered feasible. Finally, it was decided to use high-dose empirical radioiodine therapy. The persistently low TSH level was deemed to be the cause of low radioiodine uptake; therefore, recombinant human thyroid-stimulating hormone (rhTSH) stimulation was planned. After two rhTSH injections (0.9 mg on two consecutive days), the patient’s TSH levels went up to 664 µIU ml^–1^. She was subsequently treated with high-dose radioiodine of 3.7 GBq (100 mci). We used a lower dose keeping in mind the proximity of the sellar lesion to the optic chiasma and the fact that the patient was currently experiencing good vision in both eyes. A tapering dose of corticosteroid was administered to avoid any potential mass effect. A post-therapy scan (Figure 4) revealed an intensely iodine-avid remnant in the thyroid bed, multiple skeletal metastasis and faint uptake in the sellar region. Iodine uptake in the sellar region again confirmed the diagnosis of metastasis. The patient was stable during admission and at the time of discharge. An endocrinologist’s opinion was sought for further management of the hypopituitarism. 6 months post radioiodine treatment, the patient was clinically stable and an early second radioiodine therapy was planned under rhTSH stimulation.

## Discussion

Most of the time, a sellar lesion represents pituitary adenoma. Other aetiologies include Rathke and arachnoidal cysts, craniopharyngiomas, benign non-pituitary tumours and inflammatory lesions.^1,2^ Metastases account for 0.3% of all sellar masses, but a rise in incidence is being observed as life expectancy in malignant diseases is increasing, along with with improvements in imaging precision. The most common site of origin in sellar metastases is the lungs (19% of all metastases) in males and breasts in females (53%).^3,4^ Metastases to the pituitary and sella from thyroid carcinoma are very rare, and often such patients have multiple other metastatic sites. In a post-mortem study, isolated involvement of the posterior pituitary was more commonly (52%) found than involvement of both lobes (27%) or the anterior pituitary alone (21%).^5^ Predilection for the posterior pituitary was explained by the direct arterial supply and larger area of contact with adjacent dura. To the best of our knowledge, only 22 cases of thyroid cancer with sella turcica/pituitary metastasis (PM) have been reported until now. Out of these, nine were from follicular carcinoma, seven were from papillary carcinoma, five were from medullary carcinoma of the thyroid and one was due to anaplastic carcinoma of the thyroid.^6^

Clinical presentation of PM from thyroid cancer is usually owing to cranial nerve involvement or mass effect on the pituitary. However, patients with PM from other cancers generally present with one or more anterior pituitary hormone deficiency and sometimes have hyperprolactinaemia and diabetes insipidus. Unlike other cases reported in the literature, in the present case, the patient did not have cranial nerve involvement or pituitary insufficiency symptoms. The only clue in this patient was the low serum TSH, ACTH and cortisol levels.

Evaluation of patients with sellar metastasis is challenging, as differentiating sellar metastasis from an adenoma on the basis of radiological investigations is sometimes difficult. Both can have sellar enlargement, deformity and erosions. PM usually shows isointense or hypointense lesions on *T*_1_ weighted images, hyperintensity on *T*_2_ weighted images and usually enhance post contrast. A more specific MRI finding in metastasis is a dumbbell-shaped intrasellar and suprasellar tumour, with a clear indentation at the level of the diaphragma sellae.

Treatment of sellar metastasis is challenging and requires a multidisciplinary approach. The various options available include surgery, radioiodine therapy, EBRT and stereotactic radiosurgery (Gamma Knife). Each approach has its own advantages and disadvantages. Close proximity to the optic chiasma and cranial nerves would make radiotherapy and radiosurgery challenging. Furthermore, the peculiar anatomical location of the pituitary and the type of blood supply make the pituitary prone to apoplexy. It has been described in a few cases with craniopharyngioma, pituitary macroadenoma and lymphocytic hypophysitis, as also with sellar metastases from various malignancies including follicular thyroid carcinoma, especially after TSH stimulation and radioiodine treatment.^7^ Moreover, in the setting of distant extensive metastases, EBRT, Gamma Knife and surgical excision are not feasible. However, rhTSH stimulation is thought to produce a less marked increase in tumour volume and appears to be the best choice for radioiodine treatment of these metastases. Filipsson et al^8^ in a pilot study demonstrated the useful role of rhTSH in patients with central hypothyroidism due to pituitary insufficiency or pituitary damage owing to other causes. However, no clear guidelines are available for the treatment of pituitary/sellar metastasis in particular. So far, radioiodine treatment with the aid of rhTSH has been reported in five such cases.^9–12^ Out of these five cases, four patients had pituitary insufficiency and one had visual symptoms along with galactorrhoea. In our case, low cortisol and ACTH levels along with non-rising TSH levels were the only manifestation of pituitary insufficiency. Considering the overall clinical context and the complications associated with surgery or Gamma Knife therapy, radioiodine treatment under rhTSH stimulation was considered as the first choice. Post-therapy scan after high-dose radioiodine therapy showed radioiodine concentration in the sellar lesion and other metastatic sites.

## Conclusions

We report a rare case of sellar metastasis from follicular carcinoma without cranial nerve involvement and with partial pituitary insufficiency. Use of rhTSH with radioiodine therapy helped in the management of this patient without any complications.

## Learning points

1. Although rare, sellar metastasis can be seen in patients with follicular carcinoma of the thyroid.
2. Metastasis to the sellar region can cause pituitary insufficiency possibly owing to vascular compromise.
3. Use of rhTSH can be critical in this clinical scenario.
4. No definite guidelines for management of patients with sellar metastasis from thyroid carcinoma are available; however, I-131 radioiodine therapy under corticosteroid cover can be used as the first-line of treatment.

## Consent

Written informed consent was obtained from the patient for publication of this case report, including accompanying images.
